# Dental health status, dentist visiting, and dental insurance of Asian immigrants in Canada

**DOI:** 10.1186/s12939-023-01863-0

**Published:** 2023-04-25

**Authors:** Qianqian Li, Yu Wang, John C. Knight, Yanqing Yi, Sara Ozbek, Matin Shariati, Peizhong Peter Wang, Yun Zhu

**Affiliations:** 1 Division of Community Health and Humanities, Faculty of Medicine, Memorial University of Newfoundland, St. John’s, NL A1B 3V6 Canada; 2 Department of Epidemiology and Biostatistics, School of Public Health, Tianjin Medical University, Tianjin, 300070 China; 3 Dalla Lana School of Public Health, University of Toronto, 155 College Street, Room 534, Toronto, ON M5T 3M7 Canada; 4 Centre for New Immigrant Wellbeing, 200-80 Acadia Ave, Markham, ON L3R 9V1 Canada

**Keywords:** Dental health, Dental care utilization, Dental health insurance, Self-perceived oral health, Asian immigrants

## Abstract

**Objective:**

This study examined the dental care utilization and self-preserved dental health of Asian immigrants relative to non-immigrants in Canada. Factors associated with oral health-related disparities between Asian immigrants and other Canadians were further examined.

**Methods:**

We analyzed 37,935 Canadian residents aged 12 years and older in the Canadian Community Health Survey 2012–2014 microdata file. Factors (e.g., demographics, socioeconomic status, lifestyles, dental insurance coverage, and year of immigration) associated with disparities in dental health (e.g., self-perceived teeth health, dental symptoms during past one month, and teeth removed due to decay in past one year) and service utilization (e.g., visiting dentist within the last three years, visiting dentist more than once per year) between Asian immigrants and other Canadians were examined using multi-variable logistic regression models.

**Results:**

The frequency of dental care utilization was significantly lower in Asian immigrants than their non-immigrant counterparts. Asian immigrants had lower self-perceived dental health, were less likely to be aware of recent dental symptoms, and more likely to report tooth extractions due to tooth decay. Low education (OR = 0.42), male gender(OR = 1.51), low household income(OR = 1.60), non-diabetes(OR = 1.87), no dental insurance(OR = 0.24), short immigration length (OR = 1.75) may discourage Asian immigrants from dental care utilization. Additionally, a perceived lack of necessity to dentist-visiting was a crucial factor accounting for the disparities in dental care uptake between Asian immigrants and non-immigrants.

**Conclusion:**

Asian immigrants showed lower dental care utilization and oral health than native-born Canadians.

**Supplementary Information:**

The online version contains supplementary material available at 10.1186/s12939-023-01863-0.

## Introduction

Canada is an immigrant-friendly nation that welcomes a significant number of newcomers each year. In 2016, 21.9% of the total population (7.5 million) were immigrants, with new immigrants representing 3.5% of the total population. Currently, more than half of new immigrants to Canada come from Asian and Pacific Rim countries (63.5%) [1, 2]. Chinese immigrants and South Asian immigrants constitute the fastest growing ethnic minority in Canada, with immigrants from mainland China having become one of the top three largest subgroups (1.8 million) [2–4].

Accumulating evidence suggests that the health and health-seeking behavior of Canadian immigrants differed from those of the native-born population. Recent immigrants were found to be on average healthier than the general Canadian population, however, studies indicate that their health appears to deteriorate over time after immigration which is known as the ‘Healthy Immigrant Effect’ [5–8].

Surprisingly, very few studies have examined the oral health of immigrants in Canada. Most researchers focused on the oral health of children and adolescents [9–12], the elderly [13, 14], Canadian immigrants as a whole [15–19]. Recently, a few studies have focused on the oral health behaviors of Canadian Asian immigrants targeting specific Asian populations, such as the Japanese immigrants [20] and the Chinese immigrants [21]. A quantitative study compared access to dental care and unmet dental care needs of Asian immigrants and European immigrants by using Canadian Healthy Measures Survey (CHMS) data from 2007 to 2009 [22]. There were three separate provincial-level studies in specific in Canada; one targeted at refugees and immigrants in Nova Scotia [23], another on Chinese immigrants in Montreal [24], and the third one on immigrants in Ontario [25]. Most studies indicated that Canadian immigrants had poor oral health than native-born populations, and that immigrants were at higher risk to under-utilize dental care systems [26, 27]. Cost barriers, lack of dental insurance, language, and cultural barriers might be predictors of limited access to dental care among immigrants [20, 21].

Previous research on the “Healthy Immigrant Effect” upon oral health has demonstrated contradictory findings, with some studies revealing that immigrants had a greater risk of oral disease than native-born people, a pattern that improves as more time spent in Canada [16, 28]; however, another study has revealed that immigrants reported increased likelihood of dental problems sometime after immigration [27]. The scarce and inconsistent literature on the patterns of oral health and dental care utilization of Asian immigrants heightens the urgency of examining and addressing the needs and health disparities of this group.

This study aimed to compare the oral health status and dentist visiting patterns of Asian immigrants with Canadian born citizens and non-Asian immigrants and to explore factors associated with disparities in dental health and dental service utilization between Asian immigrants and other Canadians.

## Methods

### Data source

This study analyzed 26,099 Canadian born, 6,767 non-Asian, and 5,069 Asian immigrant participants aged 12 years and older in the Canadian Community Health Survey (CCHS) 2012–2014 microdata file. The CCHS is a cross-sectional survey conducted by Statistics Canada that contains various health-related details of the Canadian population. Each cycle of the CCHS involved interviews of a sample of approximately 130,000 respondents and produces an annual microdata file combining two years of data. The target population of CCHS conducted in 2012, 2013 and 2014 covers 12 years of age and over, who lived in any provinces or territories. Eligible respondents were interviewed one-on-one using computer-assisted personal interviews (CAPI) by randomly selecting households. A stratified, three-phase sample was used in 10 provinces, and in order to be representative of the covered population, survey weights provided by Statistics Canada was used in calculations and statistical analysis [29]. The study analyzed CCHS 2012–2014 microdata file data through Statistics Canada’s Research Data Center (RDC) program at Memorial University.

Based on the CCHS 2012–2014 questionnaires, Asian immigrants were defined as anyone who was born outside of Canada; not born as Canadian citizen; who were immigrants; their racial origin was Asian, which included Korean, Filipino, Japanese, Chinese, South Asian (East Indian, Pakistani or Sri Lankan), South East Asian (Cambodian or Indonesian), Laotian, Vietnamese, Arab, or West Asian (Afghani or Iranian).

In this analysis, respondents answered survey questions concerning socioeconomics, health status, dental insurances, dentist visiting and dental health status. Respondents with missing data for any questions were excluded from this analysis. Questions on race and official languages did not apply to Canadian-born residences.

### Outcome variables

Outcome variables included (1) dental health status, represented in three dimensions: self-perceived dental health status, any dental symptoms during the last one month, and teeth removed due to decay in the past 12-months; and (2) dental care utilization, comprised two variables: time since last visit to the dentist and frequency of visiting dentist. Relevant questions and options in the questionnaires are listed in Supplementary Tables 1 and 2.

### Independent variables

In this study, the main independent variable of interest was immigrant status. Immigrant status was categorized as ‘Canadian born resident’, ‘non-Asian immigrant’, ‘Asian long-term immigrant’, and ‘Asian recent immigrant’. Canadian born residents were defined as people who were born in Canada; Asian immigrants were defined as people who were born outside of Canada, and were not born as Canadian citizens, that held an immigration status in Canada during the time of the study. Ethnic origins were from the following categories: Korean, Filipino, Japanese, Chinese, East Indian, Pakistani, Sri Lankan, Cambodian, Indonesian, Laotian, Vietnamese, Arab, Afghani, or Iranian. The long-term Asian immigrants were those who had lived in Canada for over 10 years, while recent Asian immigrants were those who had lived in Canada for less than 10 years.

Covariates of interest included demographics (e.g., age, gender, marital status, and official language), socioeconomic status (e.g., education attainment, household income, employment status, and dental insurance), health status (e.g., diabetes), and lifestyle factors (e.g. smoking, alcohol consumption, and frequency of brushing teeth).

### Statistical analysis

Simple descriptive analyses were performed to describe and compare the socioeconomic characteristics, demographic characteristics, lifestyle characteristics, and prevalence of diabetes between native-born Canadians and subgroups of longtime Asian immigrants and recent Asian immigrants. The Chi square test was used to compare the percentages of people with different characteristics between Canadian-born residences and non-Asian immigrants and subgroups of recent and long-term Asian immigrants. The rescaled weights instead of original sample weight were used in the corresponding analyses to address the unequal probabilities of selection of survey respondents. The rescaled weights were calculated by dividing the original weight by the average of the original weights for the sampled units contributing to the estimator in question [29, 30]. Some studies also used the same rescaled weights in analyses of combined multiple cycles of CCHS data set [31–34]. It should be noted that the combined samples are not representative of the population of one single cycle at one point in time but rather a combined population of Canadian who participated in the CCHS over time [35]. Furthermore, a series of multivariate logistic regression model analyses were conducted to compare the dental health status and dentist visiting frequency of Asian immigrants as a whole group, as well as subgroups, with native-born Canadians after adjusting for demographic variables, socioeconomic variables, and lifestyle factors. The variances were estimated using the bootstrap weights provided by Statistics Canada [36]. Statistical significance was measured at the 95% confidence interval level. The statistical analyses were performed using SAS software package version 9.4 (SAS Institute Inc., Cary NC).

## Results

The study population consisted of 26,099 Canadian born, 6,767 non-Asian, and 5,069 Asian immigrant participants (1,937 recent Asian immigrants and 3,131 long-term Asian immigrants) aged 12 years and older (Table 1). The Canadian born population was evenly distributed among different age groups, while the majority of non-Asian immigrants were more concentrated in the senior age bracket. Asian immigrants were typically much younger than the Canadian born residents. The age distribution of Asian immigrants differed with length of residence in Canada. Recent Asian immigrants were mainly composed of persons aged 20–49 years, but most of long-term Asian immigrants were over 40 years of age. Although the three groups had similar patterns of marital status, educational attainment, and knowledge of official languages, slight differences emerged within socio-economic status. Asian Immigrants had slightly higher levels of education attainment and higher marriage rate. Immigrants had a lower level of household income compared with native born counterparts.

**Table 1:** Distribution of selected demographic, socio-economic characteristics and lifestyle in household population aged 12 or older, by immigrant status

Characteristic	Canadian born residences (n = 26,099) (%) #	Non-Asian immigrants (n = 6767) (%)#	Asian immigrants (n = 5069) (%)#	Recent Asian immigrants (n = 1937) (%)#	Long-term Asian immigrants (n = 3131) (%)#	Overall Canadian (n = 37,935) (%)#
**Age**						
12–19	13.09	4.44	8.02	13.68	4.52^E^	10.87
20–29	19.45	9.27	16.46	24.47	11.50	17.24
30–39	15.07	15.50	20.21	29.77	14.30	15.84
40–49	16.11	17.33	24.67	21.11	26.87	17.47
50–59	17.75	19.53	15.89	5.63^ F^	22.23	17.82
60-high	18.54	33.93	14.76	5.35^ F^	20.58	20.78
**Sex**						
Male	48.86	49.67	48.94	47.62	49.75	49.02
Female	51.14	50.32	51.06	52.38	50.25	50.98
**Marital status**						
Married/Common-law	53.95	66.87	69.53	64.53	72.62	58.34
Divorced/Separated/Widowed	10.35	15.49	5.73	2.73^E^	7.58	10.65
Single/Never married	35.70	17.64	24.74	32.74	19.80	31.01
**Education(household)**						
Less than secondary	17.37	16.53	14.36	15.51	13.64	16.82
Secondary/ Some secondary	26.64	25.29	22.72	23.20	22.42	25.88
Post-secondary	55.99	58.18	62.92	61.29	63.93	57.31
**Household income**						
≤$39,999	18.68	27.05	25.78	31.81	22.05	21.12
$40,000–79,999	30.47	32.66	39.26	41.96	37.59	32.03
≥$80,000	50.86	40.30	34.96	26.23	40.36	46.85
**Smoking status**						
Current smoker	20.32	13.90	8.97	10.04	8.31	17.66
Non-smoker	79.68	86.10	91.03	89.96	91.69	82.34
**Diabetes**						
Yes	5.35	7.56	7.76	3.06^E^	10.66	6.07
No	94.65	92.44	92.24	96.94	89.34	93.93
**Heavy drinker**						
Yes	15.18	6.54	2.64^E^	2.36^ F^	2.81^E^	11.96
No	84.82	93.46	97.36	97.64	97.19	88.04
**Knowledge of official languages**						
Either English/French	NA	95.62	93.62	91.25	95.09	29.57
Neither English/French	NA	4.38	6.38^E^	8.75^E^	4.91^E^	1.63
**Frequency of brush teeth**						
≥ 2/day	79.39	82.20	86.64	83.35	88.68	80.86
< 2/day	20.54	17.59	13.36	16.65	11.32	19.05
Others‡	0.07^E^	0.21^ F^	0	0	0	0.09

Data source: Canadian Community Healthy Survey annual data 2012,2013,2014

(%) #All percentages are probability weighted

n = weighted sample size. (Note: All data are weighted by the rescaled weights. The average of the rescaled weights being 1, many of the data would be fractions)

Abbreviations: NA, not applicable

E, Coefficient of variation between 16.6% and 33.3%. Estimates are considered marginal and associated with high sampling variability

F, Coefficient of variation greater than 33.3%, estimate suppressed

‡Others included don’t know/refusal/not stated

Additionally, compared with Canadian born groups (20.32%) and non-Asian immigrants (13.9%), Asian immigrants (8.97%) had a lower prevalence of current smokers (Table 1). Among Asian immigrants, 10.04% of recent Asian immigrants and 8.31% of long-term immigrants were current smokers. With respect to alcohol consumption, 15.18% of Canadian born residents were heavy drinkers and very few of the respondents in the immigrant population were heavy drinkers, including only 2.64% in Asian immigrants and 6.54% in the non-Asian immigrants. The prevalence of diabetes in long-term Asian immigrants (10.66%) surpassed that of non-Asian immigrants (7.56%) and native-born Canadian population (5.35%), even though only 3.06% of recent Asian immigrants had diabetes. All respondents kept good oral hygiene routines and there were no apparent differences among the three population groups. More than 79% of respondents from all the three groups brushed their teeth more than twice per day.

Canadian born residents (72.91%) had a significantly higher rate of dental insurance coverage than immigrants (60.66% in non-Asian immigrants, 59.51% in Asian immigrants) (Supplementary Table 3). The percentage of dental insurance coverage for immigrants rose with increased length of residence in Canada, from 52.98% in recent Asian immigrants to 63.55% in long-term Asian immigrants. Employee sponsored dental insurance accounted for most of the dental insurance coverage (> 82%). However, there was no significant difference in dental insurance types between Canadian born residents and immigrants.

With respect to dental health care utilization, Canadian born citizens were more likely to have visited the dentist at least once in the past 12-months (79.03%), compared to 72.17% of non-Asian immigrants and 63.52% of all Asian immigrants (Supplementary Table 4). Long-term Asian immigrants (69.83%) were more likely to consult a dentist once per year than recent Asian immigrants (53.33%). Results were similar for dentist-visiting behavior within the last 3 years (Supplementary Table 4), with 90.89% of native-born Canadians visiting the dentist within the last three years, compared to 88.80% of non-Asian immigrants and 84.31% of all Asian immigrants.

With respect to dental health care utilization, Asian immigrants as whole (OR = 0.59; 95% CI 0.49–0.71) along with recent Asian immigrants (OR = 0.44; 95% CI 0.32–0.59) were less likely than Canadian born residents to visit the dentist more than once per year (Table 2). Asian immigrants as whole (OR = 0.68; 95% CI 0.54–0.86) and recent Asian immigrants (OR = 0.61; 95% CI 0.43–0.87) were less likely to visit the dentist in the past three years compared to Canadian born residents. However, when looking at dental visits in the past three years, there was no statistical differences between non-Asian immigrants and Canadian born participants.

**Table 2:** Odds ratios for the association between immigrant status and dental health care utilization, household aged 12 and older

	Unadjusted OR	Age-adjusted OR	Adjusted OR‡	Adjusted OR§
**Visiting dentist within the last 3 years** (Yes)				
Canadian born residences†	1.00	1.00	1.00	1.00
Non-Asian immigrants	0.79(0.67–0.94)**	0.83(0.69–0.98)*	0.91(0.76–1.09)	0.98(0.82–1.17)
Asian immigrants	0.53(0.43–0.64)**	0.54(0.45–0.66)**	0.59(0.48–0.73)**	0.68(0.54–0.86)**
Recent Asian immigrants	0.43(0.32–0.59)**	0.46(0.33–0.62)**	0.52(0.37–0.72)**	0.61(0.43–0.87)**
Long-term Asian immigrants	0.60(0.47–0.76)**	0.61(0.48–0.78)**	0.65(0.50–0.84)**	0.73(0.55–0.97)*
**Visiting dentist more than once per year** (Yes)				
Canadian born residences†	1.00	1.00	1.00	1.00
Non-Asian immigrants	0.69(0.61–0.77)**	0.69(0.61–0.77)**	0.78(0.68–0.89)**	0.84(0.73–0.96)*
Asian immigrants	0.46(0.40–0.54)**	0.46(0.40–0.54)**	0.51(0.43–0.61)**	0.59(0.49–0.71)**
Recent Asian immigrants	0.30(0.23–0.39)**	0.31(0.24–0.40)**	0.37(0.28–0.49)**	0.44(0.32–0.59)**
Long-term Asian immigrants	0.61(0.51–0.74)**	0.60(0.50–0.73)**	0.64(0.52–0.78)**	0.73(0.58–0.93)**

Data source: Combined Canadian Community Healthy Survey annual data of 2012, 2013, and 2014

†Reference group

Note: All data are weighted by the rescaled weights. The average of the rescaled weights being 1, many of the data would be fractions.

*Significantly different from Canadian born residences (P < 0.05), using bootstrap.

**Highly significant different from Canadian born residences (P < 0.01), using bootstrap.

Abbreviations: OR, odds ratio.

‡ Adjusted for age, sex, marital status, education, household income, diabetes status, smoking status, alcohol consumption and knowledge of official language.

§ Adjusted for all factors which include age, sex, marital status, official language, education, household income, diabetes status, smoking status, alcohol consumption, knowledge of official language, self-perceived dental status, dental symptoms, and dental insurance coverage.

Table 3 displays risk factors associated with dental visiting behavior. Low education (less than secondary vs. secondary OR = 0.42), male gender (female vs. male OR = 1.51), low household income (≥ 80,000 vs. 40,000–79,999 OR = 1.60), diabetes (no vs. yes OR = 1.87), lack of dental insurance (no vs. yes OR = 0.24), short immigration length (≥ 11 years vs. ≤10 years OR = 1.75) may discourage Asian immigrants from dental care utilization.

**Table 3:** Associations of dentist visiting behavior with demographic, socioeconomic, and health-related factors, stratified by immigrant status

Characteristic	Odds Ratio 95% (Confidential interval) ‡		
	Visiting dentist more than once per year (Yes)		
	Canadian born residences	Non-Asian immigrants	Asian immigrants
**Education**			
Secondary/ Some secondary†	1.00	1.00	1.00
Less than secondary	0.71(0.60–0.85)**	1.13(0.77–1.65)	0.42(0.22–0.82)**
Post-secondary degree	1.36(1.19–1.55)**	1.63(1.14–2.32)**	1.18(0.79–1.75)
**Age**			
20–29†	1.00	1.00	1.00
12–19	3.61(2.82–4.63)**	4.25(1.95–9.24)**	3.19(1.48–6.87)**
30–39	1.06(0.88–1.28)	1.01(0.60–1.72)	0.96(0.56–1.67)
40–49	1.22(0.99–1.52)	1.71(0.98–2.98)	1.93(1.00-3.71)*
50–59	1.73(1.41–2.12)**	2.67(1.56–4.56)**	1.42(0.67–3.03)
60-high	2.16(1.75–2.65)**	3.88(2.32–6.48)**	1.97(1.01–3.85)*
**Sex**			
Male†	1.00	1.00	1.00
Female	1.41(1.26–1.58)**	1.57(1.21–2.06)**	1.51(1.06–2.16)*
**Household income**			
40,000–79,999†	1.00	1.00	1.00
80,000-more	1.80(1.59–2.05)**	1.61(1.12–2.31)**	1.60(1.07–2.39)*
No income-39,999	0.67(0.58–0.77)**	0.54(0.40–0.72)**	0.68(0.45–1.02)
**Marital status**			
Married/Common-law†	1.00	1.00	1.00
Single/Never married	1.19(1.00-1.40)*	0.75(0.49–1.14)	1.06(0.63–1.76)
Widowed/Separated/Divorced	1.08(0.91–1.27)	0.76(0.51–1.15)	0.88(0.51–1.51)
**Smoking status**			
Smoker†	1.00	1.00	1.00
No smoker	1.60(1.40–1.83)**	0.77(0.54–1.10)	1.31(0.71–2.43)
**Alcohol drinker**			
Non-heavy drink†	1.00	1.00	1.00
Heavy drink	0.94(0.80–1.10)	1.02(0.58–1.82)	1.07(0.46–2.48)
**Diabetes status**			
Yes†	1.00	1.00	1.00
No	1.17(0.96–1.43)	1.07(0.73–1.57)	1.87(1.03–3.41)*
**Dental insurance coverage**			
Yes†	1.00	1.00	1.00
No	0.29(0.26–0.33)**	0.21(0.16–0.28)**	0.24(0.17–0.33)**
**Dental symptoms**			
Yes†	1.00	1.00	1.00
No	0.94(0.84–1.05)	1.06(0.81–1.39)	1.05(0.74–1.50)
**Self-preserved dental health**			
Fair & poor†	1.00	1.00	1.00
Excellent/very good/good	3.59(3.14–4.10)**	2.90(2.16–3.89)**	1.33 (0.87–2.03)
**Language**			
English/French†	NA	1.00	1.00
Neither English nor French	NA	0.61(0.32–1.15)	0.55(0.21–1.42)
**Immigration length**			
0–10 years†	NA	NA	1.00
11-high years	NA	NA	1.75(1.17–2.61)**

Data source: Combined Canadian Community Healthy Survey annual data of 2012, 2013, and 2014

Note: All data are weighted by the rescaled weights. The average of the rescaled weights being 1, many of the data would be fractions.

*Significantly different from Canadian born residences (P < 0.05), using bootstrap.

**Highly significant different from Canadian born residences (P < 0.01), using bootstrap.

†Reference category.

‡ Adjusted for age, marital status, education, household income, diabetes status, smoking status, alcohol consumption, knowledge of official language, immigration length, self-perceived dental health, dental symptoms, and dental insurance coverage.

Interactions of frequency of dentist visiting were found with socioeconomic and lifestyle factors among Canadian born groups and non-Asian immigrants (Table 3). First, household income showed a significant association with dentist visiting behaviors among Canadian-born population and non-Asian immigrants. People with higher income were more likely to visit the dentist more than once per year. Second, people in Canadian-born groups and non-Asian immigrants who claimed an excellent/very good/good self-preserved dental health usually visited the dentist more than once per year than those in the same groups that claimed a poor/fair self-preserved dental health. However, self-preserved dental health did not show significant association with dentist visiting behaviors among Asian immigrants. Third, smoking habits were only associated with dentist-visiting behavior of Canadian-born residents but not that of other ethnic groups. Canadian-born smokers tended to consult with a dentist more often than corresponding nonsmokers. However, diabetes status was only associated with dentist-visiting behavior in Asian immigrants. Asian immigrants living with non-diabetes were almost twice as likely (OR = 1.87; 95% CI 1.03–3.41) to visit the dentist than diabetic Asian immigrants. Additionally, language did not affect the behavior of dentist visiting among the immigrant population. Finally, time since immigration was significantly associated with Asian immigrant’s dentist visiting within one year (OR = 1.75; 95% CI 1.17–2.61). Long-term Asian immigrants were more likely to consult a dentist within a year than recent Asian immigrants living in Canada.

The main reason for not visiting the dentist within the last three years were asked in the survey (Table 4). There were four main reasons identified. ‘Cost’ was the biggest reason for native born Canadians (37.07%) and non-Asian immigrants (34.80%). The next two reasons were that ‘respondent did not think it was necessary’ and ‘other’. However, there were significant differences in the response between Asian immigrants and Canadian born residents, with 64.47% of Asian immigrants agreeing that the ‘respondent did not think it was necessary’. The cost was the reason for only 20.21% of the Asian participants. In the subgroup of Asian immigrants, 71.01% of recent arrivals did not think it was necessary to visit a dentist. With increased length of stay in Canada, the proportion of immigrants agreeing that the ‘respondent did not think it was necessary’ decreased to 59.13%.

**Table 4:** Reasons for not visiting dentist within the last 3 years of household population aged 12 or older, by immigrant status

	Canadian born residences (n = 2378) (%)#†	Non-Asian immigrants (n = 757) (%)#	Asian immigrants (n = 794) (%)#	Recent Asian immigrants (n = 357) (%)#	Long-term Asian immigrants (n = 438) (%)#
			P < 0.01 **	P < 0.01 **	P < 0.01 **
Cost§	37.07	34.80	20.21^E^	16.69^E^	23.07^E^
Resp. did not think necessary§	25.41	35.40	64.47	71.01	59.13
Other reasons‡§	21.08	16.59	11.79^E^	6.78^ F^	15.88^E^
Haven’t got around it§	16.44	13.22^E^	3.53^ F^	5.52^ F^	1.92^ F^

†, Reference group

(%) # All percentages are probability weighted

Note: All data are weighted by the rescaled weights. The average of the rescaled weights being 1, many of the data would be fractions.

* Significantly different from Canadian born residences (p < 0.05), using bootstrap.

** Highly significant different from Canadian born residences (p < 0.01), using bootstrap.

E Coefficient of variation between 16.6% and 33.3%. Estimates are considered marginal and associated with high sampling variability.

F Coefficient of variation greater than 33.3%, estimate suppressed.

§ Responses are not mutually exclusive.

‡ Included Haven’t got around to it, dentist did not think necessary, personal/family responsibilities, not available when request dentist, not available in area, transportation problems, did not know where to go, fear, not specified, other (not specified).

Supplementary Table 5 shows the prevalence of self-perceived dental health, dental symptoms and teeth lost by immigrant status. Most participants rated their dental health as excellent/very good/good. with 86.84% of non-immigrants being more likely than immigrants to report their dental health status as excellent/very good/good, compared to 83.65% of non-Asian immigrants and 80.52% of Asian immigrants reported having a good/excellent dental health. Both recent and long-term Asian immigrants were more likely to report their health status as either fair or poor than non-immigrants. In the analysis by length of residence in Canada, 82.09% of recent Asian immigrants were more likely to rate their dental health status as good or excellent than 79.54% of long-term Asian immigrants.

The prevalence of any dental symptoms during the past month was similar between Asian immigrants and Canadian born residents, even when conducting analysis by length of residence in Canada. Both recent and long-term Asian immigrants reported similar rates of any dental symptoms to Canadian born residents. Non-Asian immigrants had a lower prevalence (40.61%) of dental symptoms compared with Canadian born residents (44.66%). However, regarding tooth removal due to decay, prevalence of teeth lost in Asian immigrants (5.79%) surpassed both the non-Asian immigrants (5.18%) and the native-born population (3.08%). Additionally, long-term Asian immigrants had a higher prevalence of teeth lost than Canadian-born residents (6.10% vs. 3.08%).

The differences in self-perceived dental health and teeth removed due to decay between immigrants and Canadian born residents persisted, after adjustment for age, sex, socioeconomic status, and lifestyle factors (Table 5). Results from both age-adjusted and socioeconomic status-adjusted regression models showed that Asian immigrants and other immigrants were more likely to rate their health as fair or poor than Canadian born residents. After adjustment for all socioeconomic and life-style factors, most significant differences in self-perceived dental health remained except that the difference between Canadian -born residents and recent Asian immigrants was no longer significant (OR = 1.23; 95% CI 0.86–1.75). Differences in rate of teeth removed due to decay between immigrants and Canadian born residents were also observed (Table 5). In adjusted and unadjusted models, both Asian immigrants and other immigrants were more likely to report teeth lost compared with Canadian born residents. Especially Asian immigrants as whole (OR = 2.03; 95% 1.51–2.73) and recent Asian immigrants (OR = 2.45; 95% CI 1.30–4.63) who had over twice the risk of reporting teeth lost compared to Canadian born residents. However, for acute dental symptoms during the past month, there was no significant difference between immigrants and Canadian born residents in adjusted or unadjusted models.

**Table 5:** Odds ratios for the association between immigrant status and selected health status indicators of household population aged 12 or older

	Unadjusted OR	Age-adjusted OR	Adjusted OR‡	Adjusted OR§
**Self-perceived teeth health (Fair/Poor)**				
Canadian born residences†	1.00	1.00	1.00	1.00
Non-Asian immigrants	1.30(1.12–1.49)**	1.20(1.04–1.38)*	1.13(0.97–1.32)	1.13(0.96–1.32)
Asian immigrants	1.61(1.32–1.95)**	1.63(1.34–1.98)**	1.59(1.30–1.94)**	1.50(1.22–1.84)**
Recent Asian immigrants	1.45(1.06–1.99)*	1.62(1.18–2.24)**	1.47(1.04–2.08)*	1.23(0.86–1.75)
Long-term Asian immigrants	1.70(1.35–2.15)**	1.63(1.29–2.07)**	1.66(1.30–2.11)**	1.67(1.29–2.17)**
**Dental symptoms in the past 1 month (Yes)**				
Canadian born residences†	1.00	1.00	1.00	1.00
Non-Asian immigrants	0.85(0.76–0.94)**	0.96(0.86–1.08)	0.96(0.86–1.08)	0.97(0.86–1.09)
Asian immigrants	1.00(0.86–1.15)	0.98(0.85–1.14)	0.98(0.85–1.14)	0.99(0.85–1.15)
Recent Asian immigrants	1.02(0.81–1.28)	0.92(0.72–1.16)	0.89(0.70–1.13)	0.88(0.69–1.13)
Long-term Asian immigrants	0.98(0.83–1.16)	1.03(0.87–1.22)	1.04(0.88–1.23)	1.06(0.89–1.25)
**Teeth removed due to decay in past 1 year (Yes)**				
Canadian born residences†	1.00	1.00	1.00	1.00
Non-Asian immigrants	1.69(1.32–2.16)**	1.37(1.07–1.77)*	1.33(1.02–1.73)*	1.36(1.05–1.77)*
Asian immigrants	1.93(1.45–2.58)**	1.94(1.45–2.60)**	1.90(1.42–2.55)**	2.03(1.51–2.73)**
Recent Asian immigrants	1.75(0.96–3.21)**	2.32(1.24–4.34)**	2.24(1.20–4.18)*	2.45(1.30–4.63)**
Long-term Asian immigrants	2.04(1.49–2.81)**	1.78(1.28–2.47)**	1.77(1.27–2.46)**	1.88(1.35–2.60)**

Data source: Combined Canadian Community Healthy Survey annual data of 2012, 2013, and 2014

Note: All data are weighted by the rescaled weights. The average of the rescaled weights being 1, many of the data would be fractions.

Abbreviations: OR = odds ratio.

†Reference group.

*Significantly different from Canadian born residences (p < 0.05), using bootstrap.

**Highly significant different from Canadian born residences (p < 0.01), using bootstrap.

‡ Adjusted for age, sex, marital status, education, household income, diabetes status, smoking status, alcohol consumption, immigration length, and knowledge of official language.

§ Adjusted for all factors which include age, sex, marital status, official language, education, household income, diabetes status, smoking status, alcohol consumption, knowledge of official language, teeth brush, visiting dentist more than once per year, and dental insurance coverage.

Multivariable logistic regression analyses stratified by socio-economic factors were performed separately for non-immigrant, non-Asian immigrants, and Asian immigrants to explore factors associated with self-perceived dental health, teeth removed due to decay, and occurrence of dental symptoms during the last month (Table 6). For both Canadian born residents and non-Asian immigrants, lower educational achievement, smoking, lower income, diabetes, and brushing teeth less than twice each day were associated with poorer dental health (Fair/Poor). Moreover, senior women who belonged to the native-born group also were more likely to rate their dental health also as fair or poor. However, none of the above factors were significantly associated with self-perceived dental health in Asian immigrants. After controlling for all other independent variables, the association between teeth removal and educational attainment, age, household income, smoke status, drinking habits, and teeth brushed remained statistically significant in Canadian-born citizens. People with some post-secondary educations were more likely to report teeth lost due to decay than those with secondary/some secondary. Furthermore, elderly born within Canada (more than 50 years old) were also more likely to have their teeth lost. On the contrary, people with higher household income, who were nonsmokers, or brushed their teeth more than twice per day, had lower risk of teeth lost due to decay. In other immigrants, there were associations between teeth removal and smoking habits and dentist visits. Non-Asian immigrants who were nonsmokers were less likely to lose their teeth than smokers. Interestingly, non-Asian immigrants who visited the dentist less than once a year were less likely to report teeth lost compared to non-Asian immigrants who visited the dentist frequently. However, none of the above factors were significantly associated with teeth lost in Asian immigrants.

**Table 6:** Stratified logistic regression of select dental status and dental issues by immigrant status, of household population age 12 and older, Canada

Characteristic	OR 95% (Confidential interval) §								
	Self-perceived dental health (Fair/Poor)			Dental symptoms during past one month (Yes)			Teeth removed due to decay (Yes)		
	Canadian born residences	Non-Asian immigrants	Asian immigrants	Canadian born residences	Non-Asian immigrants	Asian immigrants	Canadian born residences	Non-Asian immigrants	Asian immigrants
**Education**									
Sec/some secondary†	1.00	1.00	1.00	1.00	1.00	1.00	1.00	1.00	1.00
Less than secondary	1.18 (0.99–1.42)	1.62 (1.11–2.34) *	0.57 (0.27–1.19)	1.06 (0.93–1.21)	0.82 (0.59–1.15)	1.31 (0.75–2.29)	1.18 (0.88–1.57)	1.47 (0.74–2.92)	1.99 (0.62–6.38)
Post-secondary degree	0.73 (0.62–0.85) **	1.22 (0.86–1.73)	0.65 (0.40–1.06)	1.04 (0.95–1.15)	1.00 (0.77–1.30)	1.09 (0.75–1.57)	0.67 (0.51–0.88) **	0.99 (0.55–1.76)	1.24 (0.58–2.69)
**Sex**									
Male†	1.00	1.00	1.00	1.00	1.00	1.00	1.00	1.00	1.00
Female	0.75 (0.66–0.86) **	1.21 (0.91–1.60)	1.00 (0.65–1.55)	1.41 (1.29–1.53) **	1.61 (1.30–1.98) **	1.17 (0.86–1.59)	0.835 (0.67–1.04)	0.88 (0.55–1.38)	1.26 (0.73–2.16)
**Age**									
20–29†	1.00	1.00	1.00	1.00	1.00	1.00	1.00	1.00	1.00
12–19	0.53 (0.39–0.71) **	1.25 (0.57–2.76)	0.81 (0.29–2.22)	0.91 (0.75–1.11)	2.36 (1.34–4.15) **	0.95 (0.50–1.80)	0.15 (0.07–0.32) **	0.19 (< 0.001 - >999.99)	0.64 (0.01–61.30)
30–39	0.99 (0.77–1.28)	0.95 (0.48–1.87)	0.96 (0.48–1.92)	0.80 (0.68–0.95) **	0.84 (0.52–1.36)	1.90 (1.10–3.29) *	1.00 (0.57–1.75)	0.805 (0.23–2.84)	1.50 (0.17–13.44)
40–49	0.97 (0.75–1.25)	1.49 (0.81–2.73)	1.58 (0.76–3.28)	0.81 (0.67–0.96) *	0.74 (0.44–1.24)	1.81 (1.04–3.14) *	1.39 (0.78–2.49)	2.53 (0.80–7.94)	1.78 (0.20–15.90)
50–59	1.72 (1.37–2.16) **	1.53 (0.90–2.61)	1.96 (0.88–4.35)	0.70 (0.60–0.83) **	0.81 (0.50–1.28)	1.87 (0.89–3.94)	2.36 (1.38–4.05) **	1.75 (0.55–5.58)	3.12 (0.35–28.27)
60-high	1.10 (0.88–1.38)	1.59 (0.92–52.74)	1.81 (0.86–3.85)	0.33 (0.28–0.39) **	0.53 (0.33–0.84) **	1.23 (0.65–2.32)	2.21 (1.27–3.86) **	1.99 (0.70–5.65)	3.96 (0.46–34.35)
**Household income**									
40,000–79,999†	1.00	1.00	1.00	1.00	1.00	1.00	1.00	1.00	1.00
80,000-more	0.63 (0.52–0.75) **	1.04 (0.70–1.54)	1.02 (0.68–1.54)	0.87 (0.78–0.96) **	1.16 (0.89–1.51)	0.99 (0.71–1.38)	0.65 (0.47–0.90) **	0.81 (0.46–1.44)	0.74 (0.38–1.44)
No income-39,999	1.32 (1.12–1.55) **	1.44 (1.04–1.99) *	1.32 (0.76–2.27)	1.26 (1.12–1.42) **	1.32 (0.98–1.78)	0.86 (0.58–1.28)	1.71 (1.34–2.18) **	1.26 (0.81–1.96)	1.28 (0.57–2.85)
**Marital status**									
Married/common-law†	1.00	1.00	1.00	1.00	1.00	1.00	1.00	1.00	1.00
Single/never married	1.19 (0.99–1.43)	0.99 (0.64–1.54)	0.84 (0.47–1.52)	0.96 (0.85–1.08)	0.82 (0.56–1.20)	1.85 (1.19–2.87) **	0.89 (0.60–1.31)	0.86 (0.37–1.95)	0.79 (0.19–3.39)
Widowed/Separated/Divorced	1.06 (0.89–1.26)	1.05 (0.74–1.51)	1.08 (0.61–1.91)	0.90 (0.79–1.03)	1.03 (0.77–1.39)	2.08 (1.28–3.36) **	0.92 (0.71–1.18)	0.93 (0.57–1.52)	0.91 (0.41–2.04)
**Smoking status**									
Smoker†	1.00	1.00	1.00	1.00	1.00	1.00	1.00	1.00	1.00
No smoker	0.42 (0.36–0.49) **	0.53 (0.38–0.74) **	1.06 (0.49–2.30)	0.84 (0.76–0.93) **	0.69 (0.50–0.95) *	0.93 (0.57–1.51)	0.44 (0.35–0.56) **	0.507 (0.28–0.91) **	0.45 (0.17–1.22)
**Alcohol drinker**									
Non heavy drinker†	1.00	1.00	1.00	1.00	1.00	1.00	1.00	1.00	1.00
Heavy drinker	0.85 (0.70–1.03)	1.40 (0.80–2.43)	1.19 (0.41–3.48)	0.98 (0.87–1.12)	1.06 (0.68–1.67)	3.03 (1.30–7.08) *	0.58 (0.42–0.80) **	0.692 (0.238–2.02)	0.19 (< 0.001->999.999)
**Diabetes status**									
Yes†	1.00	1.00	1.00	1.00	1.00	1.00	1.00	1.00	1.00
No	0.75 (0.58–0.96) *	0.44 (0.31–0.64) **	0.81 (0.43–1.53)	1.013 (0.86–1.20)	0.80 (0.56–1.15)	0.84 (0.50–1.39)	0.74 (0.54–1.02)	0.89 (0.49–1.62)	0.53 (0.22–1.27)
**Teeth brush**									
< 2 a day†	1.00	1.00	1.00	1.00	1.00	1.00	1.00	1.00	1.00
≥ 2 a day	0.54 (0.48–0.62) **	0.59 (0.42–0.82) **	0.88 (0.54–1.42)	0.761 (0.69–0.84) **	0.77 (0.58–1.017)	0.99 (0.63–1.54)	0.74 (0.58–0.95) *	1.07 (0.62–1.83)	0.81 (0.40–1.66)
**Dental insurance coverage**	1.00	1.00	1.00	1.00	1.00	1.00	1.00	1.00	1.00
Yes†	1.00	1.00	1.00	1.00	1.00	1.00	1.00	1.00	1.00
No	1.09 (0.95–1.24)	1.09 (0.79–1.49)	1.52 (0.99–2.32)	0.96 (0.87–1.05)	1.00 (0.77–1.29)	0.83 (0.61–1.13)	0.81 (0.63–1.026)	1.26 (0.81–1.97)	0.65 (0.30–1.44)
**Visiting dentist more than once per year**									
Yes †	1.00	1.00	1.00	1.00	1.00	1.00	1.00	1.00	1.00
No	3.34 (2.92–3.83) **	2.83 (2.15–3.72) **	1.35 (0.90–2.03)	1.13 (1.01–1.26) **	1.27 (0.99–1.64)	1.12 (0.81–1.57)	0.89 (0.68–1.17)	0.47 (0.31–0.73) **	0.86 (0.43–1.73)
**Language**									
Either English or French†	NA	1.00	1.00	NA	1.00	1.00	NA	1.00	1.00
Neither English nor French	NA	1.56 (0.78–3.12)	2.24 (0.93–5.42)	NA	0.55 (0.32–0.95) *	1.68 (0.75–3.77)	NA	1.27 (0.51–3.18)	0.96 (0.25–3.72)
**Immigrants’ years**									
0–10 years†	NA	NA	1.00	NA	NA	1.00	NA	NA	1.00
11-high years	NA	NA	1.04 (0.66–1.63)	NA	NA	0.93 (0.67–1.29)	NA	NA	0.69 (0.30–1.57)

Data source: Combined Canadian Community Healthy Survey annual data of 2012, 2013, and 2014

Note: All data are weighted by the rescaled weights. The average of the rescaled weights being 1, many of the data would be fractions.

†Reference group.

Abbreviations: OR, odds ratio, NA: not applicable.

*Significantly different from Canadian born residences (P < 0.05), using bootstrap.

**Highly significant different from Canadian born residences (P < 0.01), using bootstrap.

OR: Odds ratio.

§ Adjusted for all factors which include immigrant status, age, sex, marital status, official language, education, household income, diabetes status, smoking status, alcohol consumption, self-perceived dental status, light dental issue, teeth brush frequency, visiting dentist more than once per year, and dental insurance coverage.

Table 6 demonstrates the unadjusted and adjusted association of individual socio demographic and physical health factors with dental symptoms. Results suggested that risk factors are different between Asian immigrant, non-Asian immigrants, and Canadian born residents for experiencing dental symptoms within the last month. In general, the factors examined in this study affected both non-Asian immigrants and Canadian born residents in similar manners for having dental symptoms. Factors such as being a female, a smoker, and within a younger age category were significantly and independently associated with high rates of acute dental symptoms in Canadian born residents and non-Asian immigrants. Moreover, Table 6 also shows that lower household income, brushing teeth less than twice per day, and lower dental visit frequency correlated with occurrence of acute dental symptoms in Canadian born residents. Nevertheless, this study showed that only age, marital status, and alcohol consumption were associated with having dental symptoms in Asian immigrants. For example, Asian immigrants who were single or widowed, fell in the age category of 30–49, and were heavy drinkers were more likely to have dental symptoms during the last month. Official language ability was not an independent predictor for dental issues or self-perceived dental status for either non-Asian or Asian immigrants.

## Discussion

In response to the oral health epidemic and unmet dental care needs, very few studies have examined these topics specifically for Asian immigrants in Canada. Most research focused on the oral health of children and adolescents [12, 37], the elderly [13, 14, 38], the Canadian immigrants in general [19, 27, 39, 40], refugees, and specific groups of immigrants in specific provinces and territories of Canada [20, 23–25], and brief descriptive summary of dental health, dental insurance and oral hygiene usage in Asian immigrants and European immigrants. This is the first study on oral health and unmet dental care needs by using large census data of the whole Asian immigrant population in Canada. It is also one of the preliminary studies focusing on comparing patterns dental care use and factor influencing Asian immigrants in Canada.

Results from this research are consistent with findings of most studies, which demonstrated that socio-economic status, dental insurance coverage, self-reported oral health, and general health behaviors are all associated with the Canadian population’s receiving recommended dental care [41–45]. With these well studied risk factors, this study also revealed that ethnicity was significantly associated with unmet dental care needs and poor dental health among the Canadian population. These findings are similar to those reported in previous literature [46–48].

Our study demonstrates the improvement of Asian immigrants’ conception related to dental care usage over time through various channels and methods. On one hand, as our study showed having lived in Canada for a longer period was associated with increased dental service use. This phenomenon of immigrant period effect on dental health practices was also observed in a previous study [14]. On the other hand, by using the latest census data, we did not find that a language barrier acted as a key barrier to receiving dental services usage as found in previous literature in the 1980s, 1990s [14] as well as in a recent study on older migrants in Canada [49].

Our study displayed that the majority of Asian immigrant participants had been accustomed to professional dental care usage. As more than 84% of Asian immigrants visited a dentist within 3 years and 63.53% participants have dentist visiting frequency more than once per year among Asian immigrants.

Although our results demonstrated Asian immigrants made good use of dental care, apparent unmet dental care needs may still exist in Asian immigrants compared with the rest of the Canadian population. With respect to whether a relationship exists between acculturation and oral behaviors, some literature reveals that a relationship exists between acculturation and oral behaviors or attitudes in Asian immigration to Canada [20]. Similarly, on one hand, this study found that well-known socio-economic and general health factors could not fully explain differences in unmet dental care needs between Asian immigrants and the Canadian population. On the other hand, when participants were asked about reasons for not visiting a dentist within the last 3 years. The majority of Asian immigrants (64.47%) stated that they did not believe dental visits were necessary, whereas Canadian-born residents cited cost as the primary reason. These indicate that a misconception about dental care usage still exists among Asian immigrants. Consistent with our findings, previous studies have noted Asian immigrants had inadequate dental health practices, which were influenced by culture. Asian immigrants may only intend to access dental care in the presence of symptoms rather than preventive oral hygiene practices, when interference may be too late and treatment is delayed [44, 50].

When it comes to dental health status, this study showed that Asian immigrants hold quite high risk (OR = 1.90, P < 0.01) of having teeth removed due to decay relative to native Canadians, even under the circumstances of having similar self-perceived dental health and dental symptoms in the Asian immigrant, non-Asian immigrants and native Canadians. Theories from other studies may offer explanations for our results. Asian immigrants’ inadequate dental health practices may lead to treatment delays [44]. Previous literature also revealed that elderly Chinese immigrants still hold misconceptions about tooth loss as they still consider tooth loss ‘normal’ and ‘undesirable’ [14, 44, 51], and they doubt that dental advice and treatments can prevent dental diseases [52]. One study shows that Asian immigrants utilize dental services in Canada based on the occurrence of symptoms rather than preventive care similar to what we found. They prefer to do self-examination or ask friends or relatives to check their teeth when symptoms show up, instead of seeking Canadian professional dental care [21]. Their results support our explanation of Asian immigrants’ dental health status, because of a lack of a professional way to effectively identify dental symptoms which may eventually turn into decay. This shows that Asian immigrants were more likely to report having teeth removed due to decay. Moreover, our results indicate socio-economic and other risk factors could not fully explain the high risk of tooth loss in Asian immigrants.

Our study results support a similar previous hypothesis that oral health beliefs and cultural values may affect care-seeking behaviors, and therefore indirectly lead to the consequence of a high risk of tooth loss in Asian immigrants. Future community health education strategies should be developed taking into account the cultural health beliefs of the Asian immigrants who are probably less likely to have knowledge about the value, function, and availability of existing professional dental care services.

Furthermore, female respondents were more likely to visit a dentist frequently within the past year. This result is consistent with other studies [19, 53]. Such gender differences in dentist visits can be explained by women’s positive attitudes towards dental visits, their better knowledge of maintaining oral health, and greater likelihood of consulting a dentist for a preventive visit [53, 54]. Future research may split the Canadian-born citizens further into those with Asian originality vs. not, which enables comparisons between Canadian-born and foreign-born Asian citizens, although migrant studies have shown that Canadian-born ethics minority have similar oral health insurance and acculturation level with the rest native-born Canadians [18, 55, 56].

## Conclusion

Our data showed that Asian immigrants visited dentist less frequently as compared to their native Canadian counterparts. Apart from pervasive socioeconomic, demographic, and health-related factors, the lack of attention paid by Asian immigrants to dental visits is related to culture. Asian immigrants may have culture to access dental care in the presence of symptoms rather than preventive oral hygiene practices. Fortunately, our results indicated that the cultural barriers to dental utilization in Asian immigrants were mitigated with the duration of stay in Canada. Moreover, Asian immigrants had lower self-perceived dental health and were less likely to be aware of recent dental symptoms compared to their non-immigrant counterparts; however, Asian immigrants were more likely than Canadian born residents to report teeth removal due to decay. Future research may investigate the factors people consider when rating self-perceived dental health. Our findings may help governments encompass understanding of the holistic concept of oral health, identifying and supporting the unique needs of the Asian immigrants’ population.

## Electronic supplementary material

Below is the link to the electronic supplementary material.

**Supplementary Table 1**: Questions of dental health status from Canadian Community Health Survey.
**Supplementary Table 2**: Questions of dental care utilization from Canadian Community Health Survey.
**Supplementary Table 3**: Rates of dental insurance coverage in household population aged 12 or older, by immigrants status.
**Supplementary Table 4**: Rate of last time visiting dentist and dentist visiting behavior per year in household population aged 12 or older, by immigrant status.
**Supplementary Table 5**: Prevalence of self-perceived dental health, dental symptoms and teeth loss in household population aged 12 or older, by immigrant status.

## Data Availability

The datasets generated and analyzed during the current study are available in the CCHS 2012–2014 microdata file data through Statistics Canada’s Research Data Center (RDC) program at Memorial University. https://www150.statcan.gc.ca/n1/en/catalogue/82M0013X.

## List of Abbreviations

CAPI: Computer Assisted Personal Interview
CCHS: Canadian Community Health Survey
CHMS: Canadian Health Measures Survey
CI: Confidence Interval
OR: Odds Ratio
RDC: Research Data Center
