# Supplementary material for: Dental health status, dentist visiting, and dental insurance of Asian immigrants in Canada
Source: Int J Equity Health. 2023 Apr 25;22:73. doi: 10.1186/s12939-023-01863-0 (PMC10131415; doi:10.1186/s12939-023-01863-0)
Supplement: Supplementary file 1 — Supplementary Table 1: Questions of dental health status from Canadian Community Health Survey. Supplementary Table 2: Questions of dental care utilization from Canadian Community Health Survey. Supplementary Table 3: Rates of dental insurance coverage in household population aged 12 or older, by immigrants status. Supplementary Table 4: Rate of last time visiting dentist and dentist visiting behavior per year in household population aged 12 or older, by immigrant status. Supplementary Table 5: Prevalence of self-perceived dental health, dental symptoms and teeth loss in household population aged 12 or older, by immigrant status. [file 12939_2023_1863_MOESM1_ESM.docx]

**International Journal for Equity in Health**

**Dental Health Status, Dentist Visiting, and Dental Insurance of Asian Immigrants in Canada**

**Supplementary Table 1**: Questions of dental health status from Canadian Community Health Survey

| **Questionnaire from CCHS** | **Answers** | **Categorized outcomes** |
| --- | --- | --- |
| “In general, would you say the health of your teeth and mouth?” | “Excellent” or “Very good” or “Good” | good oral health status |
|  | “Fair” or “Poor” | poor oral health status |
| “In the past month, have you had: a toothache?” or “In the past month, were your teeth: sensitive to hot or cold food or drinks?” or “In the past month, have you had: pain in or around the jaw joints?” or “In the past month, have you had: other pain in the mouth or face?” or “In the past month, have you had: bleeding gums?” | Either question was answered “yes” by a respondent | Had dental symptoms during past one month |
|  | All questions were answered “No” by a respondent | Do not had dental symptoms during past one month |
| “In the past 12 months, have you had any teeth removed by a dentist?” | “Yes” | Had teeth removed due to decay in past one year |
|  | “No” | Did not have teeth removed due to decay in past one year |

**Supplementary Table 2**: Questions of dental care utilization from Canadian Community Health Survey

| **Questionnaire from CCHS** | **Answers** | **Categorized outcomes** |
| --- | --- | --- |
| “When was the last time that you went to a dentist?” | “Less than 1 year ago” or “1 year to less than 2 years ago” or “1 year to less than 3 years ago” | Visiting dentist within the last 3 years (Yes) |
|  | “3 year to less than 4 years ago” or “4 year to less than 5 years ago” or “5 or more years ago” | Visiting dentist within the last 3 years (No) |
| "Do you usually visit dentist” | “More than once a year for check-ups” or “about once a year for check-ups” | Visiting dentist more than once per year (Yes) |
|  | “Less than once a year for check-ups” or “only for emergency care” | Visiting dentist more than once per year (No) |

**Supplementary Table 3**: Rates of dental insurance coverage in household population aged 12 or older, by immigrants status

|  | Canadian born residences  (n=26099)  (%)#† | Non-Asian immigrants  (n=6767)  (%)# | Asian immigrants  (n=5069)  (%)# | Recent Asian immigrants  (n=1937)  (%)# | Long-term Asian immigrants  (n=3131)  (%)# |
| --- | --- | --- | --- | --- | --- |
| Dental insurance coverage | | | | | |
|  |  | P<0.01 ** | P<0.01 ** | P<0.01 ** | P<0.01 ** |
| yes | 72.91 | 60.66 | 59.51 | 52.98 | 63.55 |
| no | 27.09 | 39.34 | 40.49 | 47.02 | 36.45 |
| Persons who have dental insurance coverage | Canadian born residences  (n= 19029)  (%)*† | Non-Asian immigrants  (n=4105)  (%)* | Asian immigrants  (n=3017)  (%)* | Recent Asian immigrants  (n= 1026)  (%)* | Long-term Asian immigrants  (n=1990)  (%)* |
| Employee sponsored dental insurance | | | | | |
| yes | 82.17 | 82.53 | 85.16 | 82.13 | 86.72 |
| no | 15.44 | 16.38 | 13.64 | 15.00^E^ | 12.93^E^ |
| Other‡ | 2.39 | 1.10^E^ | 1.21 | 2.87^F^ | 0.35^F^ |

Data source: Canadian Community Healthy Survey annual data 2012,2013,2014

(%) #All percentages are probability weighted

n=weighted sample size (Note: All data are weighted by the rescaled weights. The average of the rescaled weights being 1, many of the data would be fractions)

†Reference group

**Highly significant different from Canadian born residences (p<0.01)

‡Included don't know/ refusal/not stated

E, Coefficient of variation between 16.6% and 33.3%. Estimates are considered marginal and associated with high sampling variability.

F, Coefficient of variation greater than 33.3%, estimate suppressed

**Supplementary Table 4**: Rate of last time visiting dentist and dentist visiting behavior per year in household population aged 12 or older, by immigrant status.

|  | Canadian born residences  (n=26099)  (%)#† | Non-Asian immigrants  (n=6767)  (%)# | Asian immigrants  (n=5069)  (%)# | Recent Asian immigrants  (n=1937)  (%)# | Long-term Asian immigrants  (n=3131)  (%)# |
| --- | --- | --- | --- | --- | --- |
| Visiting dentist within the last 3 years | | | | | |
|  |  | P<0.01 ** | P<0.01 ** | P<0.01** | P<0.01** |
| yes | 90.89 | 88.80 | 84.31 | 81.58 | 85.99 |
| no and others‡ | 9.11 | 11.20 | 15.69 | 18.42 | 14.01 |
| Visiting dentist more than once per year | | | | | |
|  |  | P<0.01 ** | P<0.01 ** | P<0.01** | P<0.01** |
| yes | 79.03 | 72.17 | 63.52 | 53.33 | 69.83 |
| no | 20.97 | 27.83 | 36.48 | 46.67 | 30.17 |

Data source: Canadian Community Healthy Survey annual data 2012,2013,2014

(%)#All percentages are probability weighted

n=weighted sample size (Note: All data are weighted by the rescaled weights. The average of the rescaled weights being 1, many of the data would be fractions)

†Reference group

**Highly significant different from Canadian born residences (p<0.01)

‡Others included don't know/ refusal/not stated

**Supplementary Table 5**: Prevalence of self-perceived dental health, dental symptoms and teeth loss in household population aged 12 or older, by immigrant status.

| Characteristic | Canadian born residences  (n=26099)  (%)#† | Non-Asian immigrants  (n=6767)  (%)# | Asian immigrants  (n=5069)  (%)# | Recent Asian immigrants  (n=1937)  (%)# | Long-term Asian immigrants  (n=3131)  (%)# |
| --- | --- | --- | --- | --- | --- |
| Self-perceived health | | | | | |
|  |  | P<0.01** | P<0.01** | P<0.05* | P<0.01** |
| Excellent/very good/good | 86.84 | 83.65 | 80.52 | 82.09 | 79.54 |
| Fair/poor/ Other‡ | 13.16 | 16.35 | 19.48 | 17.91 | 20.46 |
| Teeth removed due to decay in past 1 year | | | | | |
|  |  | P<0.01** | P<0.01** |  | P<0.01** |
| Yes | 3.08 | 5.18 | 6.01 | 5.28 | 6.10 |
| No/not visit dentist/ Other‡ | 96.92 | 94.82 | 93.99 | 94.72 | 93.90 |
| Dental symptoms past 1 month | | | | | |
|  |  | P<0.05* |  |  |  |
| Yes^&^ | 44.66 | 40.61 | 44.53 | 45.13 | 44.16 |
| No/ Other‡ | 55.34 | 59.39 | 55.47 | 54.87 | 55.84 |

Data source: Combined Canadian Community Healthy Survey annual data of 2012, 2013, and 2014

(%) # All percentages are probability weighted

Note: All data are weighted by the rescaled weights. The average of the rescaled weights being 1, many of the data would be fractions

† Reference group.

* Significantly different from Canadian born residences (p<0.05), using bootstrap

** Highly significant different from Canadian born residences (p<0.01), using bootstrap

‡ Others included don't know/ refusal/not stated

& Include had a toothache, teeth sensitive to hot or cold, pain in jaw joints, pain in mouth or face, bleeding gum. Responses are not mutually exclusive
